# Orthokeratinized odontogenic cyst (OOC): Clinicopathological and radiological features of a series of 10 cases

**DOI:** 10.1186/s13000-019-0801-9

**Published:** 2019-04-04

**Authors:** Nasir Uddin, Maha Zubair, Jamshid Abdul-Ghafar, Zia Ullah Khan, Zubair Ahmad

**Affiliations:** 1 Department of Pathology and Laboratory Medicine, Aga Khan University Hospital, Karachi, Pakistan; 2 Department of Pathology and Laboratory Medicine, French Medical Institute for Mothers and Children (FMIC), Kabul, Afghanistan; 3 College of Medicine, Ziauddin University, Karachi, Pakistan

**Keywords:** Orthokeratinized odontogenic cyst, Odontogenic cyst, Odontogenic keratocyst, Keratocystic odontogenic tumor

## Abstract

**Background:**

Orthokeratinized Odontogenic Cyst (OOC) is a rare, developmental odontogenic cyst which was considered in the past to be a variant of Odontogenic keratocyst (OKC) later renamed as keratocystic odontogenic tumor (KCOT). The treatment of OOC is by enucleation and the prognosis, following enucleation is excellent with a recurrence rate of less than 2%. On the other hand, OKC has a recurrence rate between 8 and 25% after enucleation. Thus it is important to differentiate between the two entities.

**Methods:**

All cases reported in our section as OOC during the period 2013 to 2018 were retrieved from the surgical pathology files and slides were reviewed by the authors. All cases which met the histological criteria for OOC were included.

**Results:**

A total of 10 cases were included. 70% patients were males, ages ranged from 23 to 60 years, with mean age of 38.9 years. 70% of cases were located in the mandible and 90% patients presented with swelling. Radiologically, 90% cases were unilocular, all were radiolucent lesions. Mean size was 4.0 cm. Histologically, all cases demonstrated the classic microscopic features. Follow-up was available in 8 patients. All were treated by enucleation. All 8 were alive with no recurrences over a follow-up period ranging from 7 to 62 months.

**Conclusions:**

OOC has a better prognosis than OKC and needs to be differentiated from OKC due to differences in treatment and prognosis. Large majority of our cases presented with swelling and occurred in the mandibles of young males. All were radiolucent and most were unilocular. All were treated by enucleation and no recurrences occurred over follow up period ranging up to 62 months. Our findings were similar to those described in other published series.

## Background

Orthokeratinized Odontogenic Cyst (OOC) is a rare, developmental odontogenic cyst which was considered in the past to be a variant of keratocystic odontogenic tumor (KCOT). In 1981, Wright identified it, owing to its different histology and relatively low recurrence rate, as Orthokeratinized variant of odontogenic keratocyst (OKC). However, it is now clear that OOC is a distinct entity. The treatment of OOC is by enucleation. Prognosis following enucleation is excellent and recurrence following enucleation has been reported in less than 2% cases. On the other hand, OKC or KCOT has a recurrence rate between 8 and 25% after enucleation and larger lesions require surgical resection. Thus it is important to differentiate between the two entities [1–6].

Herein, we describe the clinicopathological features of 10 cases of OOC and provide a review of recent published literature.

## Materials and methods

The Surgical Pathology files of the Section of Histopathology Department of Pathology and Laboratory Medicine, Aga Khan University Hospital were searched for cases of OOC reported between 2013 and 2018. The histological slides were reviewed by two of the principal authors (NU and ZA). Cases which showed an uninflamed fibrous wall lined by thin, regular stratified, completely orthokeratinized and non-corrugated squamous epithelium with a thick, lamellated keratin layer extending into the lumen and with a granular layer extending throughout the epithelial length were included in the study. Cases with a corrugated keratin surface or inflamed fibrous wall were excluded. Radiological films of all cases were reviewed and correlated with histological findings. Informed consent was obtained in patients in whom follow up was available.

## Results

### Clinical and demographic findings

A total of 10 cases were identified and included in the study. Out of 10 patients, 7 (70%) were males and 3 (30%) were females. Male to female ratio was 2.3:1. Ages of the patients ranged from 23 to 60 years. Mean and median age was 38.9 and 35 years respectively. Of the 10 cases, 7 (70%) were located in the mandible while 3 (30%) were located in the maxilla, and 9 out of 10 patients (90%) presented with swelling and pus like discharge from the mandible or maxilla.

### Imaging findings

**Fig. 1:**
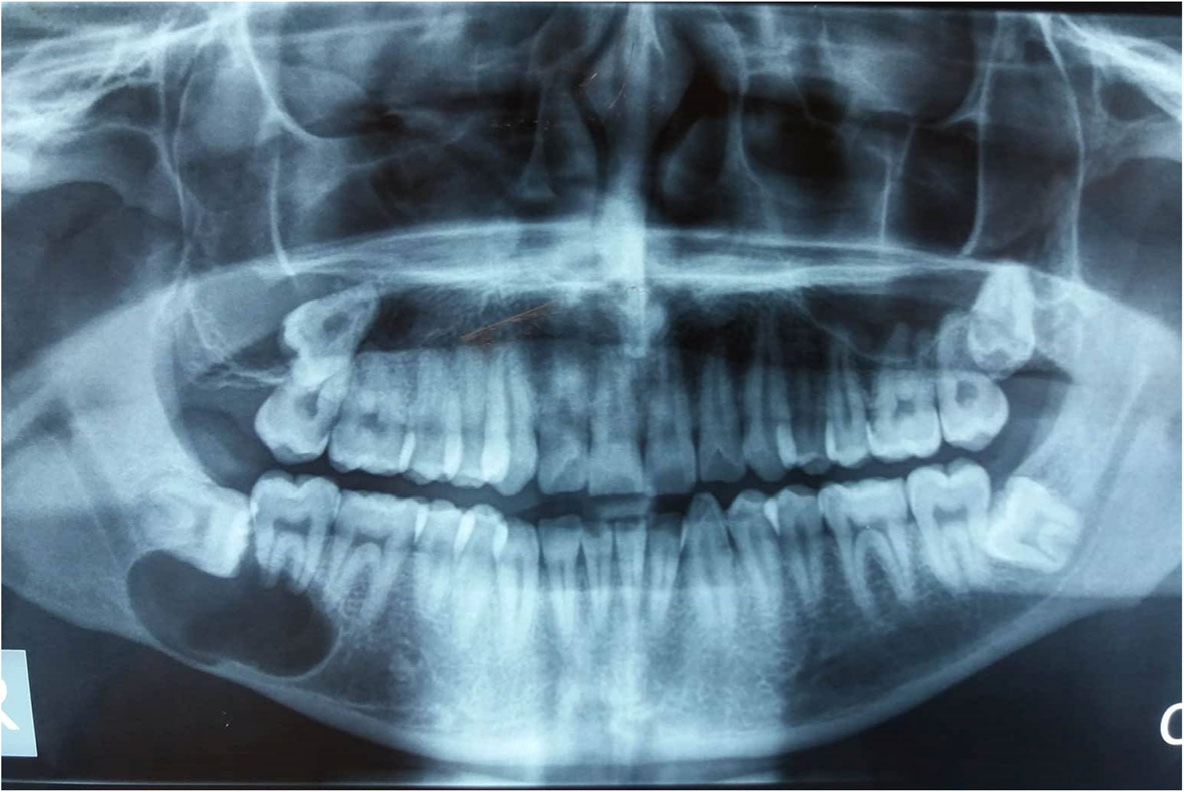
Panoramic view shows a well-demarcated unilocular radiolucency in right angle of mandible. Impacted 3rd molar tooth is seen superiorly

Radiologically, 9 out of 10 cases (90%) showed well demarcated, unilocular, radiolucent lesions (Fig. 1). However, one case was multilocular. In 3 cases (37.5%), impacted tooth was seen and 7 out of 10 cases (70%) were found in the posterior regions.

### Gross findings

All 10 patients underwent enucleation of the cyst and in all cases; the cysts were received in multiple pieces. Size of the cystic pieces received for histological examination ranged from 1.5 to 8.5 cm in aggregate with mean and median size of 4 and 3.5 cm respectively. In all cases, the pieces were flattened, irregular, soft to firm in consistency and pearl white to grey white to tan brown in color.

### Histological findings

**Fig. 2:**
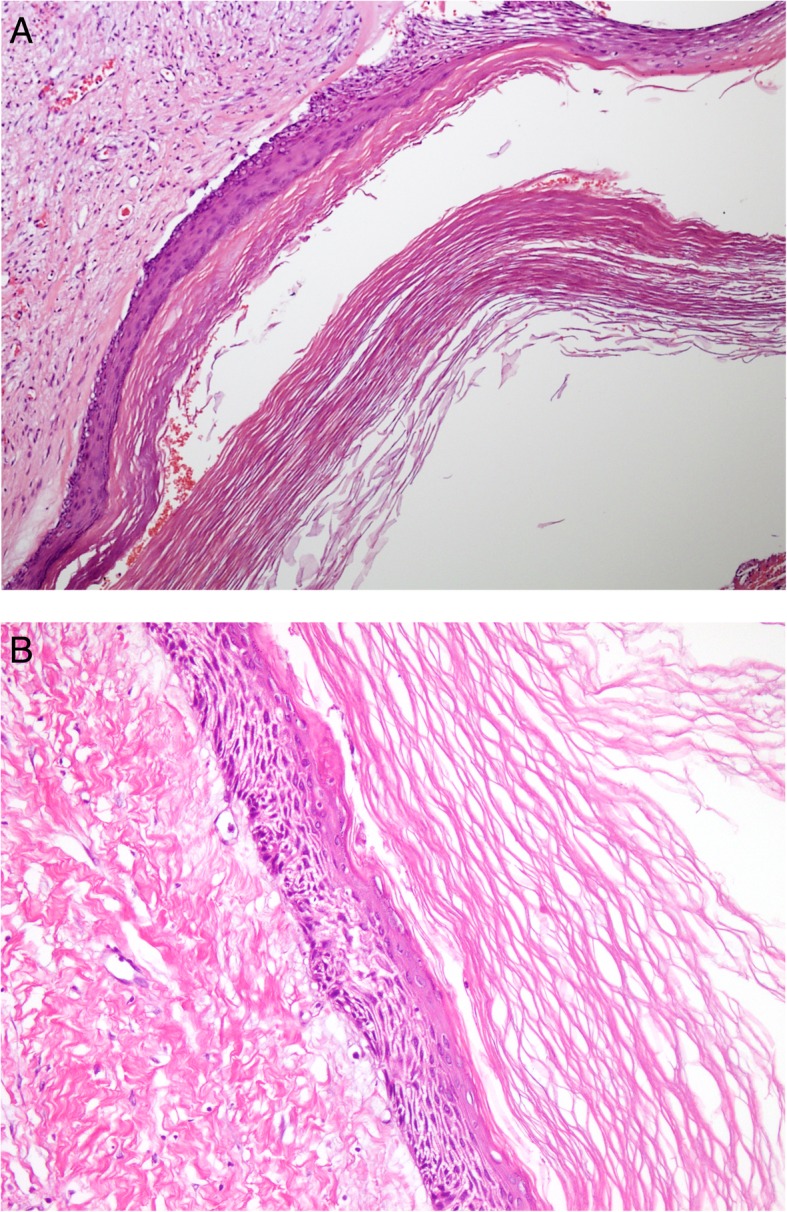
**a**) Low power shows cyst lumen is filled with lamellated keratin flakes. **b**) high power shows several layers thick epithelium with orthokeratinization. No basal layer palisading or luminal corrugation noted

Histological examination in all 10 cases showed cystic lesions lined by a thin and regular layer of stratified squamous epithelium with orthokeratinization on the surface and prominent granular cell layer. The keratin surface was thick and lamellated and did not show corrugation. Basal layer was composed in most cases of flat to cuboidal cells and palisading was not seen (Fig. 2a, b).

Focal parakeratosis was seen in three cases (30%) while sloughed keratin was seen in four cases (40%). The underlying stroma was fibrocollagenous in all cases with scattered congested blood vessels. Focal minimal chronic nonspecific inflammatory infiltrate was seen in the stroma in 7 cases (70%).

### Follow up

Follow up was available in 8 out of 10 cases. Follow up periods in these patients ranged from 7 months to 62 months. All 8 patients were treated by enucleation. All 8 patients were alive and well with no recurrences. None of the patients received any additional treatment. (Table 1).

**Table 1:** Comparison with other published series

S. No.	Year of publication	Study	Number of Cases	Age Range (years)	Mean Age (years)	Male	Female	Mandible	Maxilla	Association with Impacted Tooth	Swelling	Pain	Radiology available	Unilocular	Multilocular	Radiolucent	Size (cm)	Enucleation	Extirpation with Segmental Ostectomy	Follow up period	Recurrence
1	1998	Li et al	15	10–56	31.5	10(66%)	5(33.3%)	14(93.3%)	1(6.6%)	7(46.6%)	10(66.6%)	5(33.3%)	15(33.3%)	14(93.3%)	1(6.6%)	15(100%)	2–7	10(66.6%)	5(33.3%)	3.5 to 12 years	None
2	2010	Dong et al	61	13–75	38.9	44 (72.1%)	17 (27.9%)	55 (90.2%)	6 (9.8%)	27/54 (50%)	46 (75.4%)	13 (21.3%)	54	47 (87%)	7 (13%)	54/54	1.6–15.5, Mean size 4.8	52 (85.2%)	9 (14.7%)	6 months to 282 months	None
3	2013	Galvan et al	3	27–73	53.6	1(33.1%)	2(66.6%)	3(100%)	–	2(66.6%)	2(66.6%)	1(33.3%)	3(100%)	3(100%)	–	3(100%)	–	3(100%)	–	9 months to 12 years	None
4	2014	Selvamani et al	11	20–46	29.1	8 (72.7%)	3 (27.3%)	9 (81.8%)	2 (18.2%)	Not known	Not known	Not known	Not Known	Not known	Not known	Not known	Not known	Not known	Not known	Not known	Not known
5	2018 (submitted for publication)	Nasir et al	10	23–60	38.9	7 (70%)	3 (30%)	7 (70%)	3 (30%)	3 (30%)	9 (90%)	6 (60%)	10 (100%)	9 (90%)	1 (10%)	10 (100%)	1.5 to 8.5 (mean size 4.0	6/6 (follow up available in 6 cases)	None	7 months to 62 months (follow up available in 8 cases)	None

## Discussion

Orthokeratinized Odontogenic Cyst was included as a separate and specific entity for the first time in the 4th Edition of the World Health Organization (WHO) Classification of Head and Neck Tumors which was published in 2017 [7]. OOC was actually first described as a dermoid cyst as far back as 1927 by Schultz [8]. However, it was not until 1981 that Wright identified OOC as an entity separate from OKC and other odontogenic cysts by describing its specific, distinct clinicopathologic features [1]. In 1998, Kitano et al. published a series of 15 cases. They compared the clinical, histological and immunohistochemical features of OOC with those of OKC. Most of their patients were young males, the lesions were solitary in all 15 cases and posterior mandible was the most common location. Of their 15 patients, 9 were treated by enucleation. None of their patients developed any recurrence over a follow up period ranging from 3 to 12 years. In our series too, majority of patients were young males, lesions were solitary in most cases, and majority were located in the mandible. All 8 patients in our series, in whom follow up was available, were treated by enucleation and none of the 8 patients developed recurrence during follow up periods ranging from 7 to 62 months. Histologically, the lining epithelium in their cases lacked the typical features of OKC and demonstrated a low proliferative index [9]. In 2002, da Silva et al. performed an immunohistochemical study on 12 cases each of OOC and OKC to compare the 2 entities. They showed that OOCs expressed cytokeratin (CK) 10 consistently while expression of CK13 and CK14 was variable. In OKCs, CK10 was expressed in the superficial keratin layer while CK14 was expressed variably in the basal and suprabasal layers. Fibronectin and collagen types I and III were expressed in a fibrillar pattern in OOC and in a nonfibrillar pattern in OKC. Their results demonstrated that the immunohistochemical (IHC) profile of OKC was compatible with a more aggressive biologic behavior, thus supporting the fact that OOCs are clinically less aggressive lesions compared to OKCs with no tendency to recur [10]. Similarly in 2004, Thosaporn et al. published a comparative study of epithelial cell proliferation (proliferation index) of various odontogenic lesions by using a novel IHC cell proliferation marker IPO-38 to determine the proliferation indices of OKC, OOC, dentigerous cysts and ameloblastomas. The IPO-38 index in ameloblastomas and OKCs was similar and was significantly higher compared to IPO-38 index in OOC. Dentigerous cysts had the lowest proliferation index. The authors thus demonstrated that proliferation index was useful in predicting the differences in biological behavior among various odontogenic lesions. Their findings demonstrated that OOC was both biologically and clinicopathologically a clinical entity distinct from OKC. They argued that OKC should be regarded as a benign tumor and OOC as a non-aggressive nonneoplastic cystic lesion [11].

The largest study to date on OOC was published in 2010. Dong et al. [6] analyzed the clinicopathologic features of 61 cases of OOC in a Chinese population. They also evaluated the immunohistochemical expression of Ki67 and p63 in the epithelial linings of 15 cases each of OOC and OKC in order to compare their proliferative activity. They noted that the latter had been reclassified as a neoplasm and designated as KCOT in the 2005 WHO classification due to their intrinsic growth potential and tendency to recur [12]. Over 72% of their patients were males and mean age of their patients was around 39 years. Over 90% of their cases occurred in the mandible, 50% were associated with impacted tooth, over 75% presented as swelling, all were radiolucent on radiological examination and 87% were unilocular. None of their cases recurred over follow up perioids ranging upto 282 months. In our series too, the majority of cases occurred in the mandible of young males, most presented with swelling and were unilocular and none recurred following enucleation over follow up periods ranging upto 62 months. Dong et al. showed that compared to KCOT, the proliferative activity, as shown by Ki67 and p63 expression was significantly lower in OOC. They argued that OOCs are clinically and pathologically distinct from KCOTs and should constitute their own specific clinical entity [6]. In 2010, MacDonald-Jankowski evaluated the principal radiological features of OOC by systematic review and showed that all OOCs were radiolucent, over 90% were unilocular and just under 70% were associated with unerupted teeth. This study also concluded that although OOCs are unlikely to recur, some do. It noted that there is lack of long term follow up in the published series and that there was little published data regarding the clinical and radiological features of OOC at the time of initial presentation [5]. In 2013, Byatnal et al. published a critical appraisal of OOC. They noted that about 75% OOCs are associated with impacted teeth, thus clinically and radiologically resembling dentigerous cyst (DC) while in regard to factors such as age of occurrence, location and immunohistochemical profile, they closely resemble KCOT. Since the biological behavior of OOC is different from KCOT, it is important to differentiate between the two. This study also noted that unlike OOC, KCOTs can occur at multiple sites, have a much higher rate of recurrence and may even progress to malignancy. It was also noted that OOCs are characterized histologically by a 4 to 8 cell layer thick orthokeratinized squamous epithelium with prominent granulosa cell layer and low cuboidal basal cells while KCOTs are characterized by thick, parakeratinized epithelium with basal cells showing typical palisading of nuclei [13]. In 2013, Galvan et al. published a series of three cases. Their findings regarding the importance of differentiating OOCs from KCOTs due to marked differences in biologic behavior were similar to other published studies. They argued that a clinical feature which helps in distinguishing between the two entities, noted by others as well, is that so far OOCs have never been known to be associated with nevoid basal cell carcinoma syndrome (NBCCS) unlike KCOTs which are commonly associated with this syndrome [2, 10, 13, 14].

In 2014, Sarvaiya et al. published a systematic review of OOC. They supported Thosaporn et al’s view [11] that OOC and KCOT may be derived from the dental lamina, and argued that OOC should always be considered in the differential diagnosis of radiolucent lesions involving impacted teeth. Using a number of cytokeratin immunohistochemical stains (CK7,10,13,17,18 and 19), they showed that OOC and KCOT were distinct from DC. CK 10 and 17 were moderately expressed in OOC and KCOT and were negative to weak positive in DC. CK 18 and 19 were expressed in DC, but were negative in OOC and KCOT. CK 7 and CK 13 were weakly positive in DC, while only CK 13 was expressed in OOC and KCOT [15]. The same year, Servato et al. reported a case of OOC presenting as a periapical lesion along with a literature review. They noted that inflammatory cysts, granulomas, fibrous scars, abcesses etc. account for most cases of periapical radiolucencies and have a much better prognosis than OOC and KCOT which are much less common. They argued that histopathological examination was mandatory to differentiate between the common inflammatory lesions which have a much better prognosis and uncommon lesions like OOC and KCOT which can behave more aggressively [16]. OOCs clinically occur as single cysts. However, in 2014, Pimpalkar et al. reported a rare case of bilateral OOCs and reviewed the literature on the bilateral occurrence of OOCs [17]. In 2014, Selvamani et al. published a study to determine the prevalence of OOC and KCOT in a South Indian sample population. They also compared the clinicopathological features of the two entities. These authors also noted that due to the aggressiveness and high recurrence rate of KCOT compared to OOC, the differentiation between them is important with respect to their treatment modalities. Their results showed that the clinical features of the two entities resembled each other but histological features were distinct [18]. Bharathi et al. also published a case report of OOC and reviewed the literature [19]. Swain et al. showed that the expression of Ki67 proliferative index, p53, p63 and bcl-2 was distinctly different in OOC and KCOT. The former showed reduced expression of all these markers reflecting a lower cellular activity and more indolent behavior [20]. Another case report was published in 2015 by Pakchoian et al. and the first case of OOC in the mandibular condylar head was reported in 2016 by Managutti et al. [21, 22]. In 2016, Shetty et al. published a case report of OOC masquerading as a dentigerous cyst which was located in the maxilla. They showed that the Ki67 index of OOC was very low [4]. Single case reports of OOC continue to be published from different regions of the world [23]. Recently the first ever case of OOC with calcification was reported by Bajpai et al. The authors noted that calcification in the form of dystrophic calcification, dentinoid and cartilage have been rarely reported in KCOT but not in OOC [3]. Another recent report described an OOC with an associated KCOT component and ghost cell pilomatricoma like keratinization and calcification in a patient with Gardner Syndrome (GS). The patient was lost to follow up. The authors note that a coincidental coexistence of OOC and KCOT could not be excluded but it was more likely that the pilomatricoma like changes in an odontogenic cyst with combined OOC and KCOT components in a background of GS could be due to molecular mechanisms common to the pathogenesis of pilomatricomas and GS [23].

## Conclusion

OOC has a better prognosis than OKC and needs to be differentiated from OKC due to differences in treatment and prognosis. 70% of our patients were young males, mean age was around 39 years, 70% cases occurred in the mandible, 90% presented with swelling, all lesions were radiolucent and 90% were unilocular. All patients were treated by enucleation and recurrences were not reported over follow up periods ranging upto 62 months. Our findings were similar to those described in other published series.

## Abbreviations

CK: Cytokeratin
DC: Dentigerous Cyst
GS: Gardner Syndrome
IHC: Immunohistochemical
KCOT: Keratocystic Odontogenic Tumor
NBCCS: Nevoid Basal Cell Carcinoma Syndrome
OKC: Odontogenic Keratocyst
OOC: Orthokeratinized Odontogenic Cyst
WHO: World Health Organization
